# A Mentored Pathway into the Sciences for Underrepresented Populations: A Newly Crafted Educational Modality for Women Exploring Career Opportunities in Science, Technology, Engineering, Mathematics, and Medicine

**DOI:** 10.1089/whr.2020.0033

**Published:** 2020-09-28

**Authors:** Sheindel Ifrah Goldfeiz, Miriam Knoll, Reshma Jagsi, Chana Weinstock, James W. Pennebaker, Linda Samuels, Daniel C. Edelman

**Affiliations:** ^1^ Jewish Orthodox Women's Medical Association, Passaic, New Jersey, USA.; ^2^ Touro College of Osteopathic Medicine, New York, New York, USA.; ^3^ Department of Radiation Oncology, John Theurer Cancer Center, an HMH-MSKCC Partnership, Hackensack, New Jersey, USA.; ^4^ Department of Radiation Oncology, Center for Bioethics and Social Sciences in Medicine, University of Michigan, Ann Arbor, Michigan, USA.; ^5^ Medical Oncology and Hematology, Baltimore, Maryland, USA.; ^6^ Regents Centennial Liberal Arts Professor of Psychology, University of Texas, Austin, Texas, USA.; ^7^ Chiropractic, Baltimore, Maryland, USA.; ^8^ Center for Cancer Research, National Cancer Institutes of Health, Bethesda, Maryland, USA.

**Keywords:** biomedical science, education, women in STEMM, intersectional groups

## Abstract

***Purpose:*** The barriers generally facing women wishing to pursue careers in the disciplines of science, technology, engineering, mathematics, and medicine (STEMM) in the United States have been well described. However, additional layers of cultural beliefs and needs may pose further obstructions to women in certain cultural subgroups who wish to enter STEMM. Recognition of the challenges faced by such subgroups is important and culturally sensitive educational and training approaches may be necessary.

***Methods:*** We therefore created a science mentoring and education program incorporating the specific requirements of our test group, young Orthodox Jewish women. Our goals were to facilitate their knowledge, skills, and attitudes to embark on a scientific career in biomedicine. Interventions were designed to target physical, intellectual, emotional, and spiritual areas of growth with each intervention crafted to the sensitivity of the women's cultural and religious backgrounds.

***Results:*** Over the course of 6 years, we enrolled 59 Orthodox Jewish women, ages 16–20 years. These women spent their summers as part of the larger Summer Internship Program (SIP) at the National Institutes of Health. They participated in cohort sizes ranging from 6 to 26 in dozens of multilevel experiences in the SIP over 6–10 weeks. Participants reported strengthening interest to pursue careers in health care-related fields. Other graduates committed to pursue careers in the general sciences, and other graduate studies.

***Conclusion:*** This unique educational platform shows promise for other intersectional groups approaching educational barriers to careers in STEMM.

## Introduction

The reality of gender bias in science, technology, engineering, mathematics, and medicine (STEMM) is an increasingly popular topic, both in its influence on admission of women into these fields of study and their ability to succeed within them. According to a 2015 study, 77% of black women and 63% of white women in STEMM-related careers reported having to provide proof of competency beyond that of men of their same skill level.^1^ Women with children specifically reported being tasked with demands beyond those expected of their men colleagues and were not provided with equal opportunities for growth.^2^ Prior research has suggested that an intersection of identities may disadvantage some women in particular.^3,4^ We believe that certain subcategories of women may face other unique barriers to entering and becoming successful in scientific careers as opposed to those faced by women in the general population.

Within mainstream Orthodox Judaism, women traditionally prioritize domestic roles and large families. However, for many Orthodox women, they also desire to use their personal strengths and talents outside of the home, which creates a perceived conflict of priorities, both for the women themselves and from the community. Particularly in STEMM fields, where there is extensive training, one's career is expected to be a priority in terms of time and commitment. This creates a conflict of interest without a clear standardized resolution. Therefore, despite inclinations and strengths in the sciences, many women may forego a science career in favor of other fields where balancing work and domestic responsibilities is more readily achievable.

There are additional complex reasons for the underrepresentation of Orthodox Jewish women in STEMM careers, including the following: (1) historical barriers where outside society was not welcoming for Jews,^5–7^ (2) cultural conflicts of disparate moral values and an independent career being incompatible with the women's important family role,^8^ and (3) practical challenges, such as religious requirements, few role models who balance career and children, and a dearth of mentored pathways.^9^ These factors necessitate finding novel systems to support Jewish religious women in STEMM fields. To that end, a distinctive experience offering support, guidance, and encouragement in biomedical science was considered for Orthodox Jewish women to address all three considerations.

Specifically, a mentored pathway into STEMM was designed utilizing summer internships at the National Institutes of Health (NIH, Bethesda, MD) facilitated through the Orthodox Jewish Women's Support Group (OJW). We sought to determine whether a supportive program with the use of integrated journaling would encourage these women to enter careers in STEMM by removing barriers, conceived or practical, unique to their demographics. The OJW was created by Daniel C. Edelman, PhD, National Cancer Institute, to integrate Orthodox Jewish women into the fields of STEMM. The OJW drew its cadre from Orthodox Jewish women's schools and provided mentored support for the selected students to experience first-hand biomedical research and health care. By these means, we sought to provide these women exposure and increased awareness of all facets of STEMM careers, while maintaining their religious identities. Although community groups exist at the NIH for other intersectional and minority groups, no other group offered this type of individualized mentorship, intensive and yet broad training, cultural support, and guidance as did the OJW. We present here the educational and cultural parameters entertained to affect a positive outcome in STEMM of this underrepresented population and “hidden diversity.”^10^

## Approach

### Applications

The intensive vetting process to be accepted into the OJW NIH Summer Internship Program (SIP) at NIH revealed to the women, most for the first time, a world of professional activities and expectations that included job applications, resumes, and cover letters. Applicants were aware of the opportunity to apply for the OJW SIP primarily through word of mouth. The applicants were vetted *via* multiple means: essay of purpose, written answers to elucidating questions regarding science background and personal strengths, in-person interviews with additional questions, and discussions among the vetting volunteers, including summer interns from previous years. Women who were experienced enough to enter a laboratory environment directly without need for preparation from the 1st-year experience were permitted to apply directly to the SIP. These women still benefited from support and mentoring provided by the OJW once they had gained internships. Following the vetting process based on the expected competencies, all applicants were then placed into three categories: primary selected interns, alternate (backup) interns, and rejected applicants.

Women accepted into the pathway were subsequently required to fill out official NIH paperwork. Federal government personnel paperwork is extensive and so that no time would be lost should a primary intern withdraw, alternate interns were also processed up to the point of submission into the NIH personnel system. The women were encouraged to keep stated deadlines; however, and importantly, when deadlines were not met, the accepted applicants were not then rejected from the internship, but rather, counseled how to meet the job prerequisites. These interventions were all part of the overarching mentoring that began even before the interns arrived at NIH. Online trainings in matters of privacy, Health Insurance Portability and Accountability Act, and laboratory safety introduced the students to professional standards and knowledge necessary to participate in a sensitive health care environment.

### Housing and travel

Interns and their parents were responsible for finding their own housing for the summer and arranging modes of transport to the NIH Bethesda campus. It is forbidden by the NIH policy for employees to assist interns with obtaining these logistical needs. Instead, interns relied upon local networks in the Jewish communities surrounding the NIH Bethesda campus for carpooling and finding boarding opportunities in culturally supportive homes.

### Goals: 1st-year program

The primary goal of the 1st-year program was to introduce the interns to the wide range of disciplines in biomedicine through exposure to both clinical and research aspects of the NIH. This in turn would help the interns gauge their budding interest in science careers for further exploration. During the 1st year of the pathway at NIH, the interns shadowed in different laboratories and clinics as volunteers as an introduction to biomedicine. Once on campus, it was clearly expected that the interns would make a personal effort to understand and diligently study medical articles and new scientific material presented through a multitude of rotations and real-world experiences. Each workday was between 7 and 9 hours long and was followed by evenings spent preparing for the next day's rotations, journaling, and conducting literature research.

Having never read scientific literature in any critical way, the women were presented with the challenge to craft a literature review from primary sources to be presented at the end of the summer as a poster presentation at the NIH SIP Poster Day. NIH library staff assisted in this endeavor by teaching and coaching the women in both reading scientific literature and utilizing several scientific research modalities such as PubMed and EndNote software. Moreover, OJW leadership and mentors assisted with topic selection and formatting in creating their professional posters. This educational effort allowed the women to choose their own scientific topics and learn about the scientific literature review process, an integral skill in any scientific field.

### Goals: SIP

Subsequent to the summer spent as volunteers, the women had the opportunity to become research trainees as part of the general SIP at the NIH, and they were typically monetarily compensated. The OJW aimed to provide the interns deeper insight and experience into a career in biomedicine, specifically through research benchwork and clinical shadowing; here we coupled the mentored pathway with the SIP. Although these subsequent internships maintained active participation with the OJW, the women had day-to-day responsibilities to their respective laboratories gaining additional training in oncological, genetic, and biology research methodologies, and had the opportunity to present their research projects during the SIP Poster Day at the culmination of summer. The NIH laboratory and clinical staff guided each student in specialized benchwork, while also allowing many of the students to experience clinical shadowing, most for the first time. This extensive crossover of clinic and benchwork is unique at the NIH, and this facet of the programming allowed students to further understand the integration of seemingly disparate biomedical fields. It also provided immediate comparison of the different modalities of biomedical research, thus allowing the women to make more rapid and informed decisions as to their career paths; this somewhat obviated the practical challenge of comparing different fields of study to reach a surer career choice.

## Activities

### Journaling and adjustment

Based on works by James W. Pennebaker,^11,12^ the OJW established a journaling modality and adjustment evaluation integrated into the summer experience. Pennebaker established that journaling allows a person to put structure to their stressful circumstances, which relaxes the body and allows for all-around improved functioning.^12^ The women were given journals to specifically chronicle their NIH experiences, and at the beginning of the summer they were briefed on the expectations of journaling once a day and the benefits of journaling.^12^ The most recommended prompts came from Jason M. Satterfield in his course from the Great Courses series. They were as follows: (1) What surprised me today? (2) What moved me today? (3) What inspired me today?^13^ The OJW leadership hoped that through journaling, the women would find more meaning in their everyday NIH experiences and reflect and learn from situations that may have been stressful or culturally shocking. Personal integration through journaling of meaningful activities and challenges was a method to process events of the summer for future review and reflection. Although the women were never required to publicly share their journals, they were encouraged to reflect on what they wrote with their mentors and friends during group discussions.

The effects of journaling and SIP activities on the women's well-being, adjustment, and positive affect from their summer were evaluated *via* written surveys given at the beginning of summer and then again at the end: one anonymous survey each for journaling and adjustment. The NIH Office of Human Subjects Research Protections deemed these surveys not human subjects research, and therefore, institutional review board approval or waiver was unnecessary. The surveys were modified from the original College Adjustment Test, a 19-response questionnaire,^11^ to account for the different cultural and nonacademic venues at NIH.

### Mentoring

Mentoring of women has been reported in the literature as a necessary component to their future success in the sciences. A survey analysis was conducted following a 1-day mentoring summit for high school women who were interested in STEM-related fields to mitigate the “leaky pipeline” of women post-graduation who intended to pursue STEM careers.^14^ This mentoring summit held by the Committee of Women in Science, Engineering, and Medicine (CWSEM) included women speakers from a wide range of stages in their careers, including department heads at large research facilities, such as Johns Hopkins and the U.S. Naval Academy, as well as women in college in the beginning stages of pursuing their career goals. Their analysis found >95% of participants agreed that having a mentor was a critical aspect to their success in entering a STEM-related field. In addition, all participants agreed that the CWSEM mentoring for just the day of the summit gave them a better understanding of barriers faced by underrepresented groups in STEM. Importantly, starting mentoring early in the women's education is necessary. A study of 998 school-aged girls (grades K-8) partaking in an online STEM mentoring program found that participants experienced increased educational endeavors as well as additional success in the sciences specifically, and especially in STEM-related activities. The women also displayed a mean growth in certainty about their career trajectories in STEM.^15^

We therefore instituted structured mentoring to provide a guided pathway for the Orthodox Jewish women to address their unique cultural and practical challenges that might be associated with deviation from traditional domestic roles expected of Orthodox Jewish women. The pathway leaders, advisors, and mentors were from the same cultural community as the young women. From the Orthodox Jewish community this included religious men leaders, women physicians, and other women professionals, as well as other NIH scientists not from the community who prioritized family values in addition to their professional roles. These mentors and advisors were available for the interns to seek guidance on religious, professional, and personal life questions. NIH mentors and instructors with little exposure to Orthodox Judaism were provided cultural awareness information, which provided insight into some of the women's religious sensitivities and laws; this allowed for optimal comfort and limited surprises for both parties.

We believe that one of the most important aspects of the OJW-mentored pathway, both for 1st-year and SIP interns, was providing leadership from a similar cultural and religious background. The senior author is from the same religious faith, Orthodox Judaism, and as an experienced scientist at the NIH had both the knowledge and background to work well in religious and secular worlds. As both the OJW founder and organizer, he guided the application process, led the weekly group discussions, and provided impromptu mentoring. To help surmount cultural barriers, the program aimed to allow the students to feel secure that all religious concerns could be addressed from within the accepted bounds of their Orthodox Jewish community. Also, trust in the OJW leader's experience with handling this unique combination of worlds enabled the group leader to show the women how to make their own religious experiences relevant to the world of science. The shared values between the leadership of the program and the Orthodox Jewish community leadership helped to alleviate any pushback from community members or rabbinical leaders who may have not been comfortable with this type of integration due to perceived risks to religious observance.

As an added dimension to the mentoring aspect of the program, the 1st-year women were each placed with NIH postdoctoral and postbaccalaureate trainees. NIH trainee women were specifically sought due to the sensitive nature of gender interactions for Orthodox Jewish women: especially important in their first major experience in the secular science world. By essay and interview, these mentors were vetted for their passion to support the next generation of biomedical women scientists. The matching process was favorable to the mentors and allowed them to choose their mentees as opposed to being assigned one. Mentees were selected based upon similar personal and scientific interests by review of their SIP applications. A presummer meeting between OJW leadership and the mentors was held to set the expectations and to clarify questions relating to the unique mentoring needs for this mentee population. SIPs, however, primarily relied upon the day-to-day mentorship provided by the laboratory's principal investigator or their designate; secondarily, OJW leadership maintained an open-door policy to allow for contact if necessary.

One additional level of mentoring was instituted at the peer level. For those interns having previously experienced the 1st-year program, we requested that they be matched up with incoming 1st years for peer-to-peer mentoring (P2P). Having P2P mentoring provided a more equitable interaction for the young women allowing for greater trust overall for the entire mentored pathway and assisted with ways to deal with all barriers faced by Orthodox Jewish women. The P2P mentoring also allowed the women to facilitate relationships beyond the summer internships and extended the reach of the P2P mentoring itself.

### Group chats

We propose that a unique element critical to the success of the OJW/NIH experience, both in the 1st-year program and SIP internships, was scheduled weekly group discussions, known as “chats.” At the chats, any issue could be addressed, and feedback garnered relating to the past week's events. This was done in a forum limited to OJW members or their supporters to emphasize the teamwork needed among the women and to learn from each other's successes and difficulties. The group discussion also allowed for assimilation of the past week's activities in a context of each woman's level of religiosity. For example, how to parse the gender separation in their cultural environment with a much more mixed interaction in their professional environment.

The sense of safety was further enhanced by creating an event reporting mechanism that was woven into the chats. From the first interview and throughout the summer, interns were encouraged to make leadership and mentors aware if they felt endangered in any way: physically, emotionally, or spiritually. This required a certain level of immediate trust to be built up at the beginning of summer, which we did through small group discussions and sharing of personal and religious ideals.

## Outcomes

### Measures

**FIG. 1.**
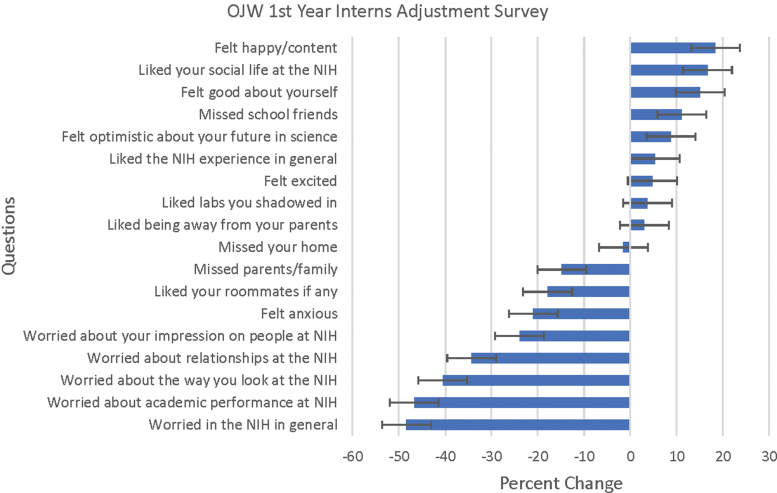
Adjustment measures for 1st-year OJW interns (*n* = 35). Women were given surveys at the beginning and conclusion of summer and asked to rate 19 questions on a scale of 1 (not at all)–7 (a great deal). In the analysis over three summers (2016–2018), the percent change in qualitative aspects of adjustment, including standard deviation, was calculated. There were no changes with feelings of loneliness (minimal to begin with, DNS) and are not represented in the chart. Depending on the question, a negative change indicated a positive affect. DNS, data not shown; NIH, National Institutes of Health; OJW, Orthodox Jewish Women's Support Group.

**FIG. 2.**
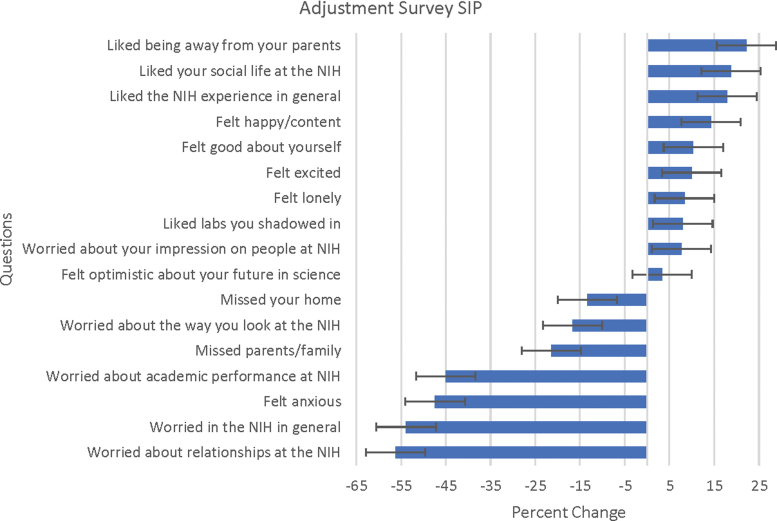
Adjustment measure for SIP interns; 2nd year of OJW and beyond (*n* = 27). Women participating in subsequent SIP internships were given the same adjustment survey as 1st-year interns at the beginning and conclusion of their summer. There was no overall change with missing friends from school and liking roommates, not represented in the chart. SIP, Summer Internship Program.

Several formal measures of student outcomes suggest a salutary impact of participation in the mentored pathway. Spanning three summers (2016–2018), the OJW leadership distributed anonymous written surveys to the interns at the beginning of summer and then again at its conclusion. They were asked to rank their emotions and beliefs surrounding the program, such as anxiety, happiness, and depression. Overall changes in these categories were analyzed. To evaluate the impacts of each subprogram, 1st-year interns and SIPs indicated their status as such on the forms. The satisfaction that the women gained from the program can be demonstrated from the increase in general knowledge, improvement of social life and self-confidence, and elevated optimism exhibited by the 1st-year interns of OJW groups (Fig. 1). These surveys showed that the gains seen in 1st-year interns appeared amplified by the experience of the subsequent SIP internships at the NIH. For example, there was a 5% increase in excitement in the 1st-year interns over the course of their summer, and yet for the SIP interns, there was a 10% increase in excitement (Fig. 2). Differentials in percent change as shown in Figure 2 showed notable increase in areas related to emotional comfort levels (14% increase in happiness/contentedness) and a positive adjustment in terms of independence (13.3% decrease in missing home) and self-confidence (10% increase).

Fifty-nine interns were enrolled and completed the mentored pathway (1st years and/or SIPs). Of those, 50 (84.7%) have returned or have wished to return to the NIH for a second summer exemplifying their desire to continue in exploring the fields of biomedical science as potential career choices. More than a third of the interns (*n* = 23, 38.9%) have returned for at least three or more summers. The 59 interns attended 15 colleges and high schools from 10 different cities and 8 states in the United States; the program design and implementation were equivalent for both groups of students.

It should be noted that two women dropped out of the program before the summers began noting financial constraints and the need for more immediate income. A third applicant who discontinued the program early shared that the time commitment would be beyond what she could dedicate to the program. Although not necessarily specific to Orthodox Jewish women, these barriers, especially that of money, do require some attention so that great minds are not lost to economic barriers.

**FIG. 3.**
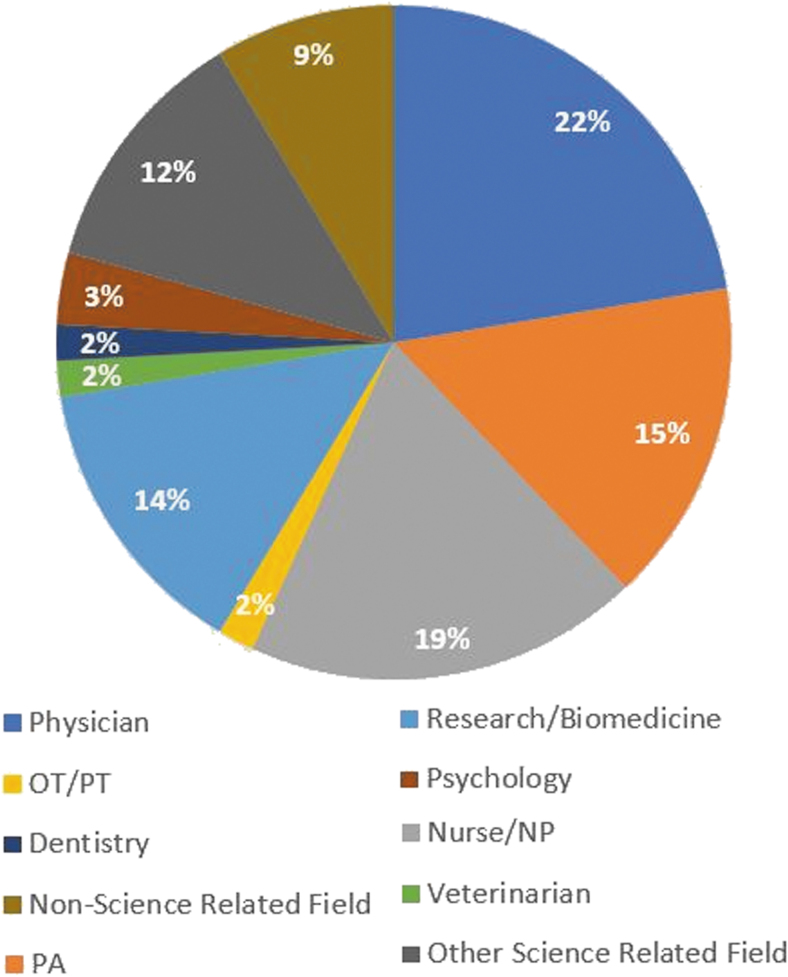
Desired careers among OJW participants. A large percentage of women (89.8%) who entered the OJW reported continued interest in entering a medical- or science-related field. Some women (10.2%) chose to enter nonscience-related fields for reasons such as extensive education and a need for an immediate income. NP, nurse practitioner; OT, occupational therapist; PA, physician's assistant; PT, physical therapist.

A large percentage of women (88.9%) who completed internships as part of the OJW reported continued interest in entering a medical- or science-related field (Fig. 3). Helpful to attaining these goals, the interns have earned standing as coauthors in eight peer-reviewed journals (Table 1),^16–23^ most with good impact factors. About 66% (39/59) of the women indicated that they wish to become medical professionals; of those, about 1 in 6 (*n* = 10) want to earn medical degrees. The remaining 29 interns expressed an interest in pursuing careers as nurses (*n* = 10), physician's assistants (PA; *n* = 12), or other health-related fields (*n* = 7). About a quarter of the interns (*n* = 15) have committed to or are pursuing careers in the general sciences, including graduate studies in engineering or bioengineering, psychology, and occupational therapy. Six women (10.2%) chose careers in fields other than science such as law, interior design, and accounting (Fig. 3). Reasons given for not electing a career related to biomedicine were reported to be the needs of extensive education and immediate income.

**Table 1.** Journals Where Orthodox Jewish Women's Support Group Interns Have Published Their Work As Coauthors

Name of journal	Impact factor
Journal of National Cancer Institute	12.589
Molecular Psychiatry	11.973
Nature Communication	11.880
PLoS Biology	8.386
Cell Reports	7.815
Diabetes	7.273
Journal of Digital Imaging	2.572
Geriatric Nursing	0.881

The research performed has been varied and published primarily in high- and medium-impact journals as indicated by respective impact factors.^16–23^

Life aptitudes we expected interns to acquire in the OJW such as critical thinking, intercultural skills, problem solving, networking skills, innovated thinking, and accountability should prove beneficial in careers other than STEMM.^24,25^ Regardless of their subsequent career paths, all the women contemplating the societal, work, and religious components of their futures were able to begin to make the necessary practical decisions about life priorities and finding one's own unique work/life balance.

Thus, we believe that the creation of this culturally sensitive mentoring pathway for young Orthodox Jewish women was successful in promoting their interest and success in pursuing STEMM careers. In relation to the typically anecdotal low rate of Orthodox Jewish women in STEMM, and the observations of increased excitement and commitment to these careers by the pathway's participants, we suggest that the program did provide meaningful value in surmounting unique barriers related to careers in science for them.

### Journaling

Anonymous surveys administered at the beginning and end of the summers (2016–2018) asked 1st-year intern participants of the OJW to rank qualitative measures regarding their journaling experience. There were insufficient survey returns from the SIP interns to draw any conclusions and it is unclear why this occurred. The leadership of OJW did not mandate participants share their entries to assure the intern's privacy, and as such were not able to conduct a qualitative analysis of journal entries; each one's journal was to be their very own safe space. A related study investigating the use of a self-reflective modality incorporated into STEM classes to benefit students underrepresented in science found that journaling helped the study groups experience positive changes in learning.^26^ In this study, self-reflective journaling was introduced at the beginning of each STEM class, and assisted students in identifying stress levels, while also allowing them to partake in peer-to-peer dialogue through sharing of journal entries. This resulted in increased positive affirmations within the class as well as in students persisting and succeeding in STEM-related coursework.

Similarly, 1st-year interns in the OJW reported, through survey analysis, an 81% increase [data not shown (DNS)] in the value they placed on the journaling aspect of their NIH experience indicating that they overcame preconceived negative notions surrounding journaling. In addition, the likelihood that the interns would share their journal entries with their mentors increased by >90% (DNS). We witnessed that when the women experienced stressful situations, impromptu sharing of their entries in group meetings allowed them to receive feedback and encouragement while also providing their peers an opportunity to glean from the experiences. When asked to estimate the impact of the program's journaling modality, students noted that journaling was mostly a positively experience at the NIH and this included how integral journaling became to them (23% increase, DNS). They also felt that there would be a long-lasting positive impact from the journaling overall (58% increase, DNS). We believe that journaling contributed to the success of the pathway because, in part, it forced the women to be mindful and aware of their daily experiences.

As hoped for, by the end of summer, the women felt increasingly more comfortable (63% increase, DNS) sharing their journal entries with friends. This may indicate that the encouragement to disclose their entries during group meetings increased their sense of trust within the group without embarrassment and made them more confident that their peers could gain valuable insight through their journal entries as well. The increased comfort with disclosing entries was also reflected in the growing likelihood that the women would share entries with their mentors (>90%, DNS). There was, however, no change in the women's disinterest in revealing their entries to their families. This could be due to the bond they formed through sharing personal events with those who experienced similar situations, which the parents and siblings had not. Additional survey studies are necessary to form a more comprehensive view of the effects of journaling and the sharing of journal entries on the OJW experience, as well as the ongoing impacts of the practice on subsequent internships at the NIH.

### Mentoring

The 1st-year women experienced emotional and social successes as measured by our survey analysis (Figs. 1 and 2). Interns reported that the mentoring provided by the OJW inspired them to think more deeply about their career paths while also connecting them with researchers whom some were able to work with in later years (personal communications). The mentored process also allowed for participants to determine and re-evaluate their life priorities. This included their choice to primarily focus on direct patient care versus scientific research studies. Importantly, as most Orthodox Jewish women are committed to begin building families during their early 20s, this gave them the opportunity to assess possibilities in a wide range of careers against the backdrop of their familial goals. For example, interns were exposed to careers where they could utilize similar skills with shorter courses of study, such as PA school. The time spent at the NIH allowed these women to explore career avenues only one small step removed from cultural expectations; they could comfortably reach outside of those norms. Alternatively, some found that indeed, they want to live a more sheltered lifestyle focused on communal religious mores with minimal secular contact. Overall, the mentored pathway taught the women how to find their personal chosen place in Orthodox Judaism with support from their peers within the context of secular society without compromising their religious beliefs and practices.

### Group chats

Discussing relevant struggles allowed the women to learn how to deal with difficult and uncomfortable situations in the workplace. The group discussion also allowed for mutual support and empathy of shared multicultural experiences. Throughout the summer, teamwork and mutual support were stressed to impart upon the women that they were not at NIH just as individuals, rather, that they have responsibilities for each other's welfare and improvement of the OJW. The support structure built into the program gave each intern a sense of community throughout the summer. It also allowed the women to network among themselves in the context of a group with shared backgrounds, values, and interests as reported through e-mail correspondence from women in the program. The chats enabled all participants in the OJW to deal actively with and prepare for the historical, cultural, and practical challenges for Orthodox Jewish women involved in STEMM fields.

## Limitations

A significant limitation in this study is the lack of qualitative analysis regarding the influence of our interventions upon the participants. Increased qualitative data analysis would be expected to further showcase the degree to which the OJW pathway impacted the women's career decisions, as well as delineate the key aspects of the program that garnered this success. Future summers should provide the means to gather these important qualitative data.

This report describes a cohort study without controls with resulting selection bias. It is unknown what the success rate would have been without our interventions; perhaps the highly motivated students who applied for this program would have pursued STEMM careers even without its support. Although we were careful to collect data on outcomes of interest, this effort was not intended to be a controlled study. However, feedback from participants was overwhelmingly positive in the survey results (Figs. 1 and 2).

## Expanded Efforts

The enacted principles and interventions can be applied in future summers at the NIH SIP integrating women with similar cultural barriers but of different faiths or other intersectional groups together with those of the Orthodox Jewish faith. We plan to incorporate discussion guidelines from the Center for Research on Learning and Teaching, University of Michigan,^27^ exchange and discuss cultural awareness sheets giving basic facts about each religion, and continually apply the principles of the Outward Mindset from the Arbinger Institute^28^ in our multiple venues of interaction.

We piloted these ideals for the summer of 2019 when a rising high school senior woman of Mormon faith joined four women of the Orthodox Jewish faith for the 1st-year experience. In brief, using our lessons learned along with cultural awareness, creating safe spaces for effective communication as well as mutually agreed upon guidelines we were successful in this integration effort as reported back to us orally and in written form by the five interns (personal communications). Although this experience only included one intern of dissimilar faith from the others, it was specifically noted by the Mormon intern in her essay to Harvard University that the program was successful because of open and honest communication. This allowed for connection and understanding *via* the group chats and the girls' shared passion for science. The Mormon intern expressed “how desire for truth and knowledge [transcends] cultural and religious differences.” She was subsequently accepted to Harvard University for undergraduate studies.

## Conclusions

We report on the success of a culturally sensitive and focused mentoring pathway into science-oriented careers. Here we targeted young women of the Orthodox Jewish faith; a group that is underrepresented in the sciences in the U.S. population. Importantly, our efforts and interventions took a holistic view of the training and educational process, incorporating a strong and active mentor/mentee relationship on multiple levels. By purposefully incorporating, acknowledging, and prioritizing the students' religious backgrounds together with scientific inquiry, we were able to successfully encourage and support their interests in pursuing STEMM careers. This framework may potentially be recreated in other unique, culturally sensitive approaches for other underrepresented groups in STEMM.

## Abbreviations Used

CWSEM: Committee of Women in Science, Engineering, and Medicine
DNS: data not shown
NIH: National Institutes of Health
OJW: Orthodox Jewish Women's Support Group
P2P: peer-to-peer mentoring
SIP: Summer Internship Program
STEMM: science, technology, engineering, mathematics, and medicine

## References

[1] Williams JC. The 5 biases pushing women out of STEM. Harvard Business Review, March 24, 2015

[2] Halley MC, Rustagi AS, Torres JS, et al. Physician mothers' experience of workplace discrimination: A qualitative analysis. BMJ 2018;363:k4926 30541926 10.1136/bmj.k4926 PMC6889631

[3] Carbado DW, Crenshaw KW, Mays VM, Tomlinson B INTERSECTIONALITY: Mapping the movements of a theory. Du Bois Rev 2013;10:303–312 25285150 10.1017/S1742058X13000349 PMC4181947

[4] Crenshaw K. Demarginalizing the intersection of race and sex: A black feminist critique of antidiscrimination doctrine, feminist theory and antiracist politics. Univ Chicago Legal Forum 1989;1989:139–167

[5] Knight P. Conspiracy theories in American history: An encyclopedia. Santa Barbara, CA: ABC-CLIO, 2003: 2

[6] Perry M, Schweitzer FM, Perry MM Anti-Semetism: Myth and hate from antiquity to present. New York, NY: Palgrave Macmillan, 2002

[7] Penslar DJ. Shylock's children: Economics and Jewish identity in modern. Europe: University of California Press, 2001

[8] Rich T. Judaism 101: Women in Judaism 1995–2011. Available at: www.jewfaq.org/women.htm Accessed 824, 2020

[9] Brin DW. Working Mom MD: Always on call. AAMC News, 2017 Available at: www.aamc.org/new-insights/working-mom-MD-always-on-call

[10] Naumburg CG. Judaism: A Hidden Diversity. Smith Coll Stud Soc Work 2007;77:21

[11] Pennebaker JW, Colder M, Sharp LK Accelerating the coping process. J Pers Soc Psychol 1990;58:528–537 2324942 10.1037//0022-3514.58.3.528

[12] Pennebaker JW, Chung CK Expressive writing and its links to mental and physical health. In: Friedman H, ed. Oxford handbook of health psychology. New York, NY: Oxford, 2011

[13] Satterfield JM. Cognitive behavioral therapy: Techniques for retraining your brain. Chantilly, VA: The Great Courses, 2015

[14] Cole M. Turning the STEM tide: An approach for mentoring young women on how to thrive in STEM careers. In: Directorate WaMR, ed. Aberdeen Proving Ground, MD: Army Research Laboratory, 2014

[15] Stoeger H, Debatin T, Heilemann M, Ziegler A. Online mentoring for talented girls in STEM: The role of relationship quality and changes in learning environments in explaining mentoring success. New Directions for Child and Adolescent Development, 2019 10.1002/cad.20320 31670457

[16] Baskin AS, Linderman JD, Brychta RJ, et al. Regulation of human adipose tissue activation, gallbladder size, and bile acid metabolism by a beta3-adrenergic receptor agonist. Diabetes 2018;67:2113–2125 29980535 10.2337/db18-0462 PMC6152342

[17] Dangelmaier E, Lazar SB, Lal A Long noncoding RNAs: p53's secret weapon in the fight against cancer? PLoS Biol 2019;17:e3000143 30759134 10.1371/journal.pbio.3000143 PMC6391031

[18] Fox ME, Chandra R, Menken MS, et al. Dendritic remodeling of D1 neurons by RhoA/Rho-kinase mediates depression-like behavior. Mol Psychiatry 2020;25:1022–1034 30120419 10.1038/s41380-018-0211-5 PMC6378138

[19] Gara SK, Lack J, Zhang L, Harris E, Cam M, Kebebew E Metastatic adrenocortical carcinoma displays higher mutation rate and tumor heterogeneity than primary tumors. Nat Commun 2018;9:4172 30301885 10.1038/s41467-018-06366-z PMC6178360

[20] Goyal N, Apolo AB, Berman ED, et al. ENABLE (Exportable Notation and Bookmark List Engine): An interface to manage tumor measurement data from PACS to cancer databases. J Digit Imaging 2017;30:275–286 28074302 10.1007/s10278-016-9938-1 PMC5422230

[21] Nilubol N, Yuan Z, Paciotti GF, et al. Novel dual-action targeted nanomedicine in mice with metastatic thyroid cancer and pancreatic neuroendocrine tumors. J Natl Cancer Inst 2018;110:1019–1029 29481652 10.1093/jnci/djy003 PMC6136933

[22] Sathyamurthy A, Johnson KR, Matson KJE, et al. Massively parallel single nucleus transcriptional profiling defines spinal cord neurons and their activity during behavior. Cell Rep 2018;22:2216–2225 29466745 10.1016/j.celrep.2018.02.003 PMC5849084

[23] Wickersham KE, Crothers M, Puth D, Weiss MN, Powell K, Resnick B Targeted therapy use in adults with cancer >/ = 85 years of age. Geriatr Nurs 2019;40:63–66 30327159 10.1016/j.gerinurse.2018.06.014

[24] Association of American Colleges and Universities and Hart Research Associates. It takes more than a major: Employer priorities for college learning and student success. Washington, DC: Association of American Colleges and Universities and Hart Research Associates, 2013

[25] Nelson KL, Rauter CM, Cutucache CE Life science undergraduate mentors in NE STEM 4U significantly outperform their peers in critical thinking skills. CBE Life Sci Educ 2018;17:ar54 30335602 10.1187/cbe.18-03-0038 PMC6755882

[26] Landhuis E. Using expressive language to keep students grounded and engaged in science courses. KQED, Inc., 2018. Available at: https://www.kqed.org/mindshift/50644/using-expressive-writing-to-keep-students-grounded-and-engaged-in-science-courses Accessed 824, 2020

[27] Center for Research on Learning and Teaching. Guidelines for classroom interactions: University of Michigan. 2020. Available at: http://crlt.umich.edu/examples-discussion-guidelines Accessed 824, 2020

[28] The Arbinger Institute. Leadership and self-deception: Getting out of the box. San Francisco, CA: Berret–Koehler Publishers, Inc., 2010

